# TiDeTree: a Bayesian phylogenetic framework to estimate single-cell trees and population dynamic parameters from genetic lineage tracing data

**DOI:** 10.1098/rspb.2022.1844

**Published:** 2022-11-09

**Authors:** Sophie Seidel, Tanja Stadler

**Affiliations:** ^1^ Department of Biosystems Science and Engineering, ETH Zürich, Basel, Switzerland; ^2^ Swiss Institute of Bioinformatics (SIB), Lausanne, Switzerland

**Keywords:** phylogenetics, lineage tracing, Bayesian inference, single cell, phylodynamics, development

## Abstract

The development of organisms and tissues is dictated by an elaborate balance between cell division, apoptosis and differentiation: the cell population dynamics. To quantify these dynamics, we propose a phylodynamic inference approach based on single-cell lineage recorder data. We developed a Bayesian phylogenetic framework—time-scaled developmental trees (TiDeTree)—that uses lineage recorder data to estimate time-scaled single-cell trees. By implementing TiDeTree within BEAST 2, we enable joint inference of the time-scaled trees and the cell population dynamics. We validated TiDeTree using simulations and showed that performance further improves when including multiple independent sources of information into the inference, such as frequencies of editing outcomes or experimental replicates. We benchmarked TiDeTree against state-of-the-art methods and show comparable performance in terms of tree topology, plus direct assessment of uncertainty and co-estimation of additional parameters. To demonstrate TiDeTree’s use in practice, we analysed a public dataset containing lineage data from approximately 100 stem cell colonies. We estimated a time-scaled phylogeny for each colony; as well as the cell division and apoptosis rates underlying the growth dynamics of all colonies. We envision that TiDeTree will find broad application in the analysis of single-cell lineage tracing data, which will improve our understanding of cellular processes during development.

## 1. Introduction

Understanding the principles of development is a major goal for developmental, regenerative and cancer biology. Cell phylogenies contain rich information on cellular events during development; they depict the ancestral relationships between cells, map the origin of cell types and contain a signal for key developmental parameters, such as the cell division, death and differentiation rates [1]. Recent developments in genetic lineage tracing provide the data to reconstruct such cell phylogenies and use them to quantify developmental processes.

Several genetic lineage tracing systems, or recorders, have been developed [2–7], all relying on an enzyme, such as CRISPR-Cas9, to edit or scar, genomic target regions that are passed on to successive generations. Hence, they provide a record of the ancestral relationships between cells and can be used to reconstruct a cell phylogeny.

To date, different computational methods exist to reconstruct cell phylogenies from lineage recorder data. Initially, methods based on maximum parsimony [2] were used and custom algorithms for cell phylogenies were developed [5]. These methods aim to reconstruct a tree that minimizes the number of edit acquisition events. However, the assumption that edit acquisition is rare is violated by recurrent editing outcomes of the CRISPR-Cas9 enzyme [8]. To reduce biased inference, frequently occurring scars had to be excluded during data pre-processing, resulting in data loss [5]. Alternatively, distance-based methods are used (such as UPGMA in TypeWriter [9]) which ignore all information beyond the pairwise distances between cells; again, some data, namely cells where not all genomic targets are sequenced, must be omitted.

Recently, methods were developed that can incorporate *a priori* information on the frequency of editing outcomes [10–12], which helps reduce biased inference due to homoplasy or the exclusive use of pairwise distances. While some approaches focus on improving scalability to reconstruct trees with millions of cells [10,13], others focus on detailed modelling of the editing process to enable more accurate inference [12]. A key example of the latter is the maximum-likelihood framework GAPML [12], which models the editing process of a GESTALT recorder [2]. Additionally, a molecular clock assumption is employed allowing to order cell division events (branching events in the phylogeny) relative to each other.

Until recently, no framework existed that can infer time-scaled trees, that is phylogenies where branch lengths are scaled in absolute duration and a time is associated with each cell division. Additionally, all existing methods only provide a single best estimate of the tree linking cells together and ignore the phylogenetic uncertainty in this estimate.

To overcome these limitations, we developed a time-dependent editing model and show how to calculate its likelihood. We implemented this model in time-scaled developmental trees (TiDeTree), a package within the BEAST 2 [14] platform. We show how to use it for Bayesian phylogenetic inference of time-scaled cell lineage trees, which enables the inference of population parameters alongside phylogenies and further provides a natural framework for quantifying uncertainty and incorporating prior information. We show how integrating commonly available additional information can result in more accurate and precise estimates. Finally, we apply TiDeTree to lineage tracing data [15] and estimate time-scaled trees and population parameters. Compared to other methods [11], TiDeTree’s ability to recover the correct tree topology is always among the top three, while also being the only method to estimate cell division times and to quantify uncertainty.

## 2. Material and methods

### (a) Phylogenetic model for lineage tracing

Here, we introduce a time-dependent editing model. Based on this model, we derive the likelihood function to perform phylogenetic inference from lineage tracing data.

#### (i) A general lineage recorder

We consider the following set-up of a lineage recorder: a precursor cell contains *m* genomic regions, henceforth called *targets*, that are targeted by an editing enzyme. Different targets can be distinguished by target-specific *barcodes*. We refer to the combined region of a target and its barcode as an *integration*.

**Figure 1.**
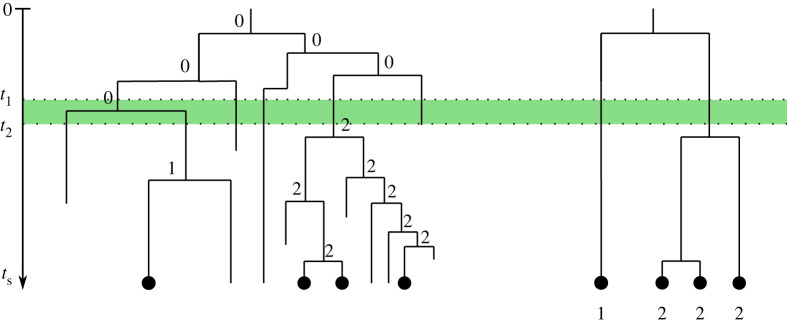
Example cell phylogeny for initial cell with one genomic target. Numbers denote the target state at each node: 0 corresponds to an unedited, 1 to a silenced and 2 to a scarred target. Targets can be silenced at any time. Scarring can only occur during the scarring window (shading). Internal nodes represent cell divisions and branch lengths the time between them. Left: complete developmental phylogeny with all cells and their target state at internal nodes. Right: reconstructed phylogeny of only the cells sampled at time *t*_*s*_. (Online version in colour.)

Given such a set-up, the experiment starts with a precursor cell where all targets are unedited (depicted as state 0 in figure 1). During a time period from *t*_1_ to *t*_2_, the *scarring window*, any target can be scarred, i.e. transition from the unedited state (0) to one of *S* scarred states (e.g. state 2 in figure 1). Experimentally, this is implemented either via injection of the editing enzyme into the precursor cell (*t*_1_ = 0) or by inducing the enzyme’s expression later during development (*t*_1_ > 0). Usually, *t*_2_ is determined by independent experiments that identify at what time point the fraction of unedited integrations stops decreasing [4,5]. Throughout the experiment, a target can be silenced at any time (state 1 in figure 1).

At the end of the experiment, at time *t*_*s*_, a subset of cells is selected for sequencing to determine the states of their integrations. Either DNA or RNA sequencing may be used, as long as the readout allows to assign integrations to a single cell. Both sequencing technologies might fail to report the presence of an integration due to dropouts during the sequencing process. Additionally, RNA sequencing cannot record an integration if the genomic region containing the integration was silenced during development.

#### (ii) Substitution model

We now formulate a model for the lineage recorder introduced in the last section. We model barcode evolution as a continuous time Markov chain with state space Ω={unedited, silenced, scarred}, initial state *X*_0_ = {unedited} and a time-dependent (piecewise-constant) transition rate matrix:

$$Q_{\left(t<t_{1}\right)}=Q_{\left(t>t_{2}\right)}=\left(\begin{array}{ccc} -l & l & 0\\ 0 & 0 & 0\\ 0 & l & -l \end{array}\right)$$

and

$$Q_{\left(t_{1}\leq t\leq t_{2}\right)}=\left(\begin{array}{ccc} -l-s & l & s\\ 0 & 0 & 0\\ 0 & l & -l \end{array}\right).$$

Silencing of a barcode’s genomic region occurs at constant rate *l* throughout the experimental period, while scarring only occurs during the scarring window, *t*_1_ ≤ *t* ≤ *t*_2_, at constant rate *s*.

Note that scarring and silencing cannot be reversed. Further, while both unedited and scarred barcodes may be silenced at any time, once silenced a barcode cannot be scarred, and once scarred cannot be scarred again.

In the following, we extend the simple substitution model above to *S* scarred states, where each state *i* corresponds to a different scarring outcome. Additionally, we introduce the editing rate *r* at which state *S* is accumulated per unit of time. We therefore set *s*_*S*_ = 1 and generally assume that any state *i* is reached at rate *r* × *s*_*i*_. Thus, the scarring rates *s*_*i*_ can be interpreted as rate multipliers that indicate how often state *i* arises relative to state *S*. Alternatively, the substitution model can be parameterized such that the editing rate *r* represents the rate of any state occurring (i.e. the molecular clock rate) as was done for the analysis of the intMEMOIR dataset (for details, see electronic supplementary material, appendix C).

In our substitution model, this leads to increasing the state space of the Markov chain to $\Omega =\{\mathrm{unedited},\;\mathrm{silenced},\;{\mathrm{scar}}_{1},$ ${\mathrm{scar}}_{2},\ldots ,\;{\mathrm{scar}}_{S}\}$

2.1 $$Q_{\left(t<t_{1}\right)}=Q_{\left(t>t_{2}\right)}=\left(\begin{array}{ccccc} -l & l & 0 & \ldots & 0\\ 0 & 0 & 0 & \ldots & 0\\ 0 & l & -l & \ldots & 0\\ \ldots & \ldots & \ldots & \ldots & \ldots \\ 0 & l & 0 & \ldots & -l \end{array}\right)$$

and

2.2 $$Q_{\left(t_{1}\leq t\leq t_{2}\right)}=\left(\begin{array}{ccccc} -l-r\times \sum_{i}s_{i} & l & r\times s_{1} & \ldots & r\times s_{S}\\ 0 & 0 & 0 & \ldots & 0\\ 0 & l & -l & \ldots & 0\\ \ldots & \ldots & \ldots & \ldots & \ldots \\ 0 & l & 0 & \ldots & -l \end{array}\right).$$

Note that this substitution model is not time reversible. Generally, time reversibility is a desirable property, because it ensures that the transition rate matrix can be diagonalized, which simplifies computation. However, the transition matrices (equations (2.1) and (2.2)) are diagonalizable and the transition probability matrix, *P*_*t*_, can be determined analytically (see electronic supplementary material, appendix). As a result, we can compute *P*_*t*_ in *O*(*k*), when matrix diagonalization would require *O*(*k*^3^), where *k* = *S* + 2 is the dimension of the rate matrix.

In experimental settings, some scarred states may occur more often than others. Especially frequently occurring scars can bias phylogenetic inference. By allowing for scar state-specific scarring rates, *s*_*i*_, we avoid this bias. Essentially, this allows us to weigh the information content a scarred state provides since, for example, a scarred state with a high scarring rate is likely to arise several times on the tree, while a scarred state with low scarring rate will probably arise only once.

#### (iii) Phylogenetic likelihood

To calculate the likelihood of the model parameters given the data (target states of the sampled cells) and the model, we introduce the following notation. Let *T* be a tree with *n* tips representing the reconstructed phylogeny of the sampled cells. This tree has *n* tip nodes of degree 1, *n* − 1 internal nodes of degree 3, including the root node, the most recent common ancestor (MRCA) of all samples. Additionally, we have an origin node of degree 1 ancestral to the root node which specifies the start of the experiment (figure 1, right). By convention, we number internal nodes from (*n* + 1) to (2*n*) from the tips towards the origin. We further subdivide all branches at time points *t*_1_ and *t*_2_ and label these additional nodes of degree 2 with 2*n* + 1, 2*n* + 2, …2*n* + *d* + 1. Further, let *τ*_*i*_ be the length of the branch that connects node *i* to its parent, *π*_*i*_.

Let *θ* summarize the parameters of the transition rate matrix, i.e. the scarring rates *s*_1_, …, *s*_*S*_, with *s*_*S*_ = 1 and the per-unit rate *r* of site *S*, and silencing rate *l*. Note that each branch is associated with one transition rate matrix (matrix in equation (2.1) or 2.2), i.e. the process does not change along a branch. We use vector *b*_*i*_ to refer to the state of all integrations in node *i* and specify with *b*_*i*,*j*_ the state of the *j*th integration for *j* ∈ 1, …, *m*. For the tips, these *b*_1_, …, *b*_*n*_ are known. Then, we can calculate the likelihood of the tree and parameters *θ*, given the target states of all *m* integrations at the sampled cells (*b*_1_, …, *b*_*n*_) by summing over all internal node states

2.3 $$\begin{array}{rl} \mathrm{Lik}(T,\theta |b_{1},\ldots ,b_{n}) & =\sum_{b_{2n}\in \Omega }\text{Pr}(b_{1},\ldots ,b_{n}|T,\theta ,b_{2n})\text{Pr}(b_{2n}|T,\theta )\\ & =\sum_{b_{2n}\in \Omega }\prod_{\;j=1}^{m}\text{Pr}(b_{1,j},\ldots ,b_{n,j}|T,\theta ,b_{2n})\text{Pr}(b_{2n}|T,\theta )\\ & =\sum_{b_{2n}\in \Omega }\prod_{\;j=1}^{m}\sum_{b_{n+1,j}\in \Omega }\ldots \sum_{b_{2n-1,j}\in \Omega }\sum_{b_{2n+1,j}\in \Omega }\ldots \sum_{b_{2n+d+1}\in \Omega }\\ & \prod_{\begin{array}{c} i=1\\ i\neq 2n \end{array}}^{2n+d+1}P_{\tau_{i}}(b_{i,j}|b_{\pi_{i},j};\theta )\text{Pr}(b_{2n,j}|T,\theta ), \end{array}$$

where $P_{t_{i}}(b_{i,j}|b_{\pi_{i},j};\theta )$ represent the transition probabilities from parent node *π*_*i*_ to its child node *i* along branch length *t*_*i*_. They are derived from the transition probability matrices that are calculated analytically (electronic supplementary material, appendix, equations (9) and (10)).

All ancestral targets, i.e. targets at the origin, are unedited (by the experimental design). Thus, the probability of the origin state being unedited (0) is

2.4 $$P(b_{2n,j}=0|T,\theta )=1\;\forall j,$$

### (b) Inference using the phylogenetic model

We implemented the substitution model and the likelihood calculation employing the pruning algorithm [16] (leading the same result as equation (2.3) but avoiding its summations) within the package time-scaled developmental trees (TiDeTree) available within the widely used BEAST 2 [14] platform, thus enabling phylogenetic and phylodynamic inference under the model described above.

While we provide the complete derivation of the likelihood calculation, our computational analyses below are focused on the scenario where no silencing occurs (i.e. *l* = 0). This allows us to directly link the results of the *in silico* analyses to the results from the experimental lineage tracing data where silencing was not present.

The set-up of the method validation and assessment of accuracy and precision are described in the electronic supplementary material, appendices A and B. We apply the method to experimental lineage tracing data using the set-up explained in the electronic supplementary material, appendix C. The results of these analyses are presented in the next section.

## 3. Results

### (a) Bayesian phylogenetic inference from lineage tracing data

**Figure 2.**
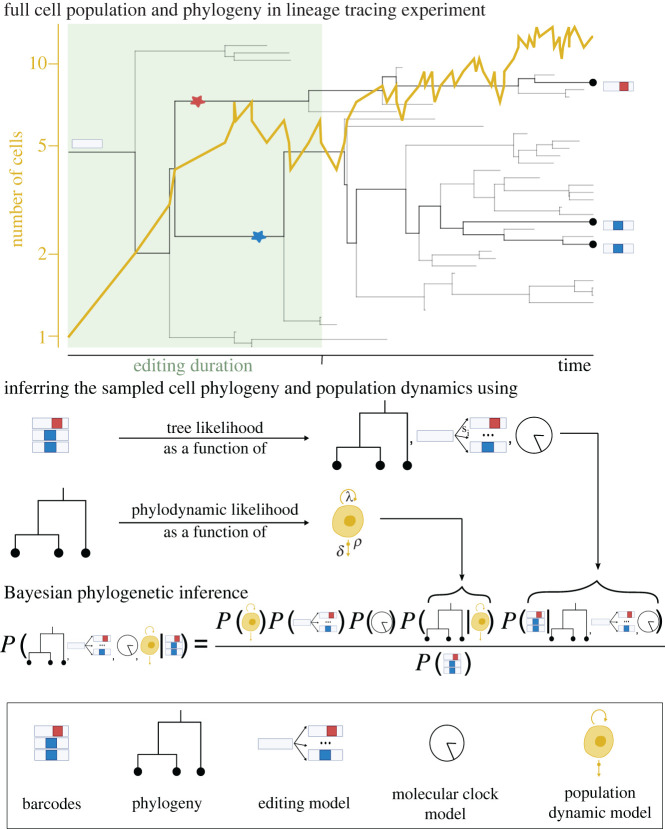
Bayesian phylogenetic inference using lineage tracing data. In a lineage tracing experiment, the full cell population process and the complete phylogeny connecting all cells are unobserved. A subset of cells is sequenced yielding the lineage tracing barcodes. We can reconstruct the sampled phylogenetic tree from the barcodes using the tree likelihood. The sampled phylogenetic tree contains information on the full cell population process—such as on cell division (*λ*) and apoptosis rates (*δ*)—that can be extracted using the phylodynamic likelihood. In Bayesian phylogenetic inference, we jointly infer the sampled phylogeny and the parameters of the cell population process. Information on any model parameter can easily be incorporated by the use of prior distributions.

In a lineage tracing experiment, the full cell population process is unobserved and the lineage barcodes are obtained from a subset of cells at a single time point (figure 2). Within a Bayesian phylogenetic inference approach, we can use the lineage barcodes to reconstruct the phylogeny of the sampled cells and the phylogeny to estimate the population dynamic parameters of the underlying cell population. We developed a time-dependent editing model and derived its likelihood function, that enables phylogenetic inference from genetic lineage tracing data in a Bayesian Markov chain Monte Carlo (MCMC) framework (detailed in 'Material and methods'). We implemented these calculations as a BEAST 2 [14] package called TiDeTree, enabling the inference of population parameters alongside the phylogenetic trees.

### (b) *In silico* validation of the implementation

We validate TiDeTree using well-calibrated simulations [17]; meaning we draw simulation parameters from a prior distribution and use them to simulate genetic sequences (see electronic supplementary material, appendix A). Then, we estimate the posterior distribution of our parameters given the sequence data and the same prior distribution. If the method is correctly implemented, 95% of the true simulation parameters are contained within the inferred 95% highest posterior density (HPD) interval. Thus, we compute the coverage for all parameters, i.e. the number of times the true parameter was contained within the 95% posterior interval. The coverages converge to 95% for all inferred parameters (electronic supplementary material, table S1) indicating correctness of the implementation.

### (c) Assessing parameter inference based on simulated data

**Figure 3.**
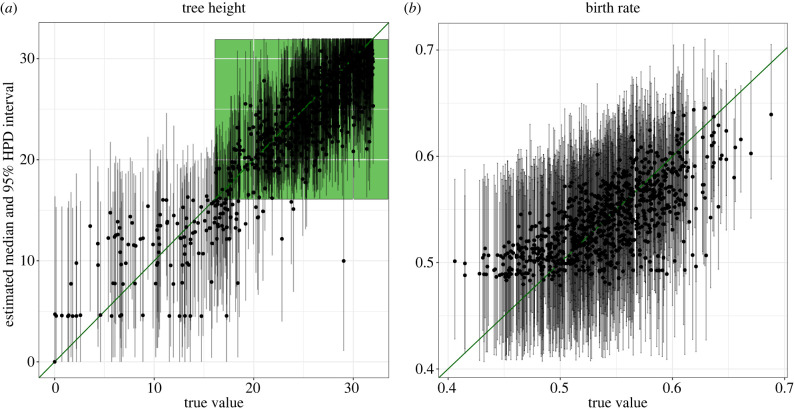
Parameter estimates based on simulated data. The graphs show the median estimates (black dots) and 95% HPD intervals (grey lines) on the *y*-axis and the true values on the *x*-axis based on 860 simulations. Two different parameters are shown: (*a*) tree height and (*b*) birth rate. The diagonal green line indicates the performance of a perfect estimator. The green shaded area in *a* illustrates the time period in which editing takes place. Thus, if the most recent common ancestor of the cells is younger than the editing period, the sequencing data does not contain any signal on the tree topology. (Online version in colour.)

To assess the TiDeTree’s capacity to correctly infer parameters from the data, we simulate sequences and trees containing up to 700 cells under a set of pre-defined simulation parameters (as opposed to drawing the simulation parameters from a distribution as before). Then we perform inference using TiDeTree to infer the trees and model parameters from the sequences (see electronic supplementary material, appendix B). We contrast the true simulation parameters against the estimated medians in figure 3 and electronic supplementary material, figure S1.

In our simulation, editing was allowed from the beginning of the simulation for 16 arbitrary time units (see green shaded area). Then, sequences were only propagated to descendant cells for another 16 time units. Hence, if the MRCA of all cells is younger than (i.e. occurs after) the editing period, all its descendants will share the same sequences. Hence, there will be no phylogenetic signal in the data. Therefore, we report the correlation between the parameter estimates and the true values separately for those datasets where the MRCA is within (phylogenetic signal) and after the editing period (no phylogenetic signal) in table 1.

**Table 1.** Correlations between parameter estimates and true values. We report Pearson’s correlation coefficient (*R*) and confidence intervals (CI) between the estimated parameters and the true values. We distinguish between datasets where the most recent common ancestor of all cells occurred within the editing period (phylogenetic signal) or after (no phylogenetic signal).

	phylogenetic signal		no phylogenetic signal	
	*R*	CI	*R*	CI
tree height	0.81	[0.79, 0.84]	0.71	[0.61, 0.8]
tree length	0.99	[0.98, 0.99]	0.92	[0.88, 0.94]
birth rate	0.72	[0.69, 0.76]	0.38	[0.21, 0.53]
sampling proportion	0.44	[0.38, 0.5]	0.45	[0.29, 0.58]

For all parameters but the sampling proportion the correlation is much stronger for datasets with phylogenetic signal compared to those without (confidence intervals non-overlapping, or only marginally overlapping for the tree height), as expected. For the sampling proportion, the presence of phylogenetic signal does not influence the correlation. In summary, based on our simulations, TiDeTree can reliably estimate the tree height, length and birth rate from sequence data with phylogenetic signal, when editing occurred for half of the experimental time span.

We further inspect how differing number of cells in the dataset influence the inference performance (electronic supplementary material, figure S2). We use the bias and the root mean square error (RMSE) to assess the accuracy and the 95% HPD interval width to assess the precision of our estimates. We find that the number of tips and the HPD width for the considered parameters (estimated tree length, tree height, sampling proportion, birth rate) are negatively correlated. The correlation is of a similar degree for tree length, tree height and sampling proportion (*R* ≈ −0.2, CIs = [− 0.25, − 0.13], [− 0.25, − 0.12], [− 0.28, 0.16], respectively) and is stronger for the birth rate (*R* = −0.74, CI = [− 0.77, − 0.71]). Hence, increasing the number of cells in the dataset leads to increased precision of the parameters of interest as expected. Regarding accuracy, the bias of all parameters is not correlated with the number of cells, but fluctuates around 0. However, the RMSE decreases for increasing cell numbers for all parameters; most strongly again for the birth rate (*R* = −0.22, CI = [− 0.28, − 0.15]) and least strongly for the sampling proportion (*R* = −0.1, CI = [− 0.17, − 0.04]). Therefore, an increased number of cells leads to greater accuracy on the parameter estimates. Finally, we track the run time until convergence for datasets with different number of cells (electronic supplementary material, S3). We find that datasets with 100, 250 and 500 cells require on average 6 h, 96 h and 400 h until convergence, respectively. In all, increasing the number of cells leads to more accurate and precise parameter estimates but also increased run time as expected.

### (d) Assessing accuracy and precision of parameter inference when integrating additional information

In a third simulation study, we assess how commonly available independent information can further improve the inference. We simulate sequences and trees from a set of simulation parameters. First, we apply TiDeTree to infer the model parameters from one sequence alignment and using weakly informative priors (scenario A). Second, we apply TiDeTree as before and assume the relative scarring rates are known (e.g. from a separate experiment as in [15] or from CRISPR screens [18–20]) (scenario B). Third, we provide the true relative scarring rates and 10 sequence alignments, mimicking 10 experimental replicates, and pool the model parameters across trees (scenario C).

We compare the tree topology across different scenarios using the posterior support: the posterior probability of the true internal nodes with heights greater than 16 time units (electronic supplementary material, figure S4). We find that the mean and minimum posterior support do not vary greatly (overlapping confidence intervals) between scenarios. Nevertheless, for scenario C, the average mean and minimum posterior support are highest as expected.

**Figure 4.**
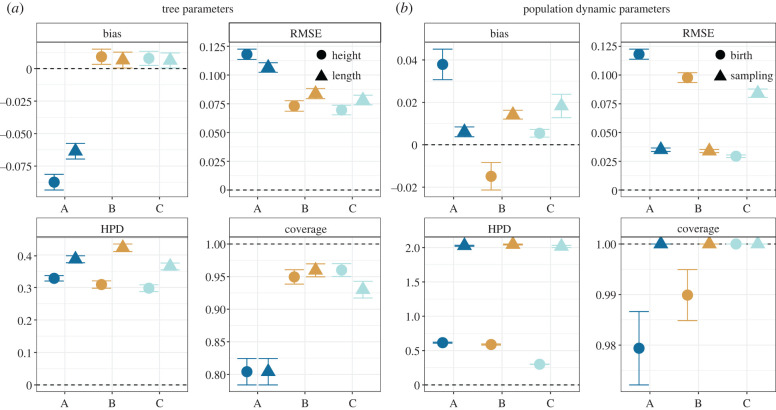
Inference performance when using different inputs. Input description: A = barcode alignment; B = barcode alignment and scarring rates; C = 10 barcode alignments from experimental replicates and scarring rates. Bias, RMSE, HPD width and coverage for the (*a*) tree height and tree length, and (*b*) birth rate and sampling proportion, based on different inputs. The error bars show the standard errors of the mean. The dashed line shows the best possible value for each metric. (Online version in colour.)

In the following, we assess the accuracy of the tree parameters (length and height) and the population dynamic parameters (birth rate and sampling proportion) (figure 4) using the bias and RMSE. Further we assess the precision using the 95% HPD width and the coverage.

For the tree parameters, the bias for scenarios B and C is 10 times and the RMSE is 1.5 times smaller compared to scenario A. Differences between B and C are insignificant (confidence intervals overlapping). This indicates that known scarring rates greatly improve the accuracy of the tree parameters, while further adding experimental replicates does not add much information about the trees. Moreover, the HPD width for the tree height decreases slightly from 0.33 ± 0.01 in scenario A to 0.30 ± 0.01 in scenario C. For the tree length, the HPD width rises from 0.39 in A to 0.42 in B to 0.36 in C. The coverage for both tree parameters increases from approximately 80% in scenario A to approximately 95% in scenarios B and C. Hence, known scarring rates increase the coverage and accuracy of the tree length and height, but do not greatly increase their precision.

For the population dynamic parameters, we observe that the bias fluctuates for different parameters and scenarios. The RMSE for the birth rate decreases continually from 0.12 ± 0.004 in A to 0.1 ± 0.004 in B and 0.029 ± 0.001 in C. The RMSE for the sampling proportion is similar for A and B (both 0.034 ± 0.0.002) while increased for scenario C (0.08 ± 0.004). Additionally, while the HPD width for the sampling proportion rate remains unchanged across inputs (2.0 ± 0.1), it becomes two times smaller for the birth rate in C (0.3 ± 0.002) compared to A and B (0.6 ± 0.01). Moreover, the coverage for the birth rate increases continually from A to C. Therefore, adding known scarring rates and experimental replicates leads to more accurate results, but only additionally adding experimental replicates results in more precise estimates.

### (e) Benchmark and application on lineage tracing data

We benchmark TiDeTree on the intMEMOIR dataset (as available during the DREAM challenge) [11,15] by comparing it against existing methods. The data are divided into a training and a test set. However, in most situations where lineage tracing is used, training datasets are not available. Hence, we use TiDeTree as an unsupervised method, i.e. we do not use the ground truth trees from the training set. Instead, we use all 106 alignments within one inference, where we reconstruct a tree for each alignment while the scarring rates and population dynamic parameters are shared across trees. Each alignment contains between 3–39 cells (electronic supplementary material, figure S5).

To estimate node heights, TiDeTree must assume a global editing rate, called a molecular clock rate in traditional phylogenetics. To test whether this assumption holds, we computed the proportion of edits at binned internal node intervals (electronic supplementary material, figure S11). The proportion of edits increases throughout the entire experimental period and the time duration up to an internal node correlates with the expected number of edits (*R* = 0.48, CI = [0.45, 0.51]). Hence, we assume a molecular clock.

We evaluate TiDeTree’s performance for three different editing model settings. In the first analysis, (a), we assume that the global editing rate and the edit-outcome rate multipliers (i.e. for acquiring an inversion or a deletion) are the same for all sites. In the second setting, (b), we allow edit-outcome probabilities to vary across sites. In the third setting, (c), we additionally allow the rate of any edit being introduced to vary across sites. In all analyses, we jointly estimate the phylogeny and the cell population’s cell division (birth) and apoptosis (death) rate.

**Figure 5.**
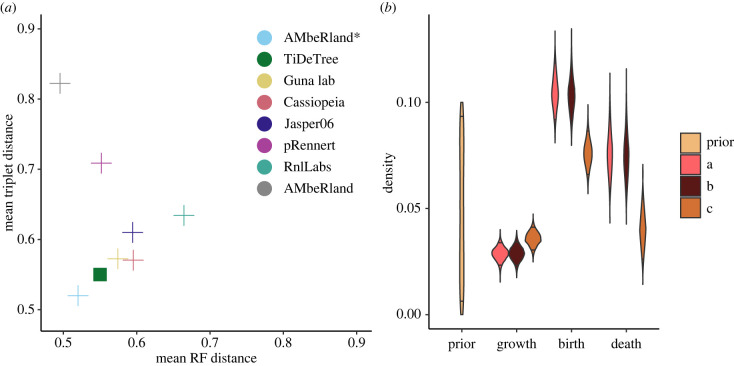
Application on lineage tracing data. (*a*) Topology benchmark ranking methods according to the Robinson–Foulds (RF) and triplet distance; TiDeTree is highlighted by a filled square. (*b*) Estimated population dynamic parameters: prior and posterior distributions of the growth rate, posterior of the birth rate and death rate. We report these estimates for the following editing model settings. (a) The rate of editing and the edit-outcome specific rates are the same for all sites. (b) The rate of editing is the same for all sites, but each site has its own edit-outcome specific rate. (c) Each site has its own rate of editing and edit-outcome specific rate. (Online version in colour.)

In terms of tree topology, TiDeTree is among the top three methods (figure 5*a*; electronic supplementary material, S6) for all editing model settings. Here, it is important to highlight that it outperforms many methods although it ignores the training set, i.e. uses less data. Moreover, in addition to a point estimate for the tree, we also report the posterior distribution of trees (electronic supplementary material, figure S9). This posterior can be visually inspected to assess which parts of the tree are well supported and which are uncertain. Further, for each tree posterior, we constructed the 95% credible set, the smallest set of trees that make up 95% of the posterior. To construct this set, unique tree topologies with highest probability are continually added to the set, until their sum reaches 95%. The exact true tree topology is contained in the credible set in 36% (for c; 31%, and 32% for a and b) of alignments. We noted that some credible sets contain >10^4^ unique tree topologies, indicating little signal in the data to reliably favour one topology over another. Upon exclusion of credible sets with >10^4^ trees, we recover the exact true tree topology within the credible set 68% (for c, 60% and 63% for a and b) of the time.

In addition to the tree topology, we estimate the tree branch lengths and the cell division (birth) and apoptosis (death) rates of the population (figure 5*b*). For editing model settings a and b, we estimate the 95% HPD interval of the birth rate to [0.09, 0.12] and of the death rate to [0.06, 0.09] per hour. For the experimental period of 54 h, the birth rate corresponds to an expected number of [4.9–6.3] cell divisions. For setting c, the estimated birth and death rates are significantly lower amounting to [0.07, 0.09] for the birth and [0.03, 0.05] for the death rate per hour. This leads to an expected number of cell divisions between [3.6–4.6], which matches the reported number of approximately 4–5 cell divisions in the associated manuscript. Thus, by allowing the editing rate to vary across sites, we can correctly estimate the population dynamic parameters. This finding can be explained as follows. In analysis c, we estimate an editing rate for each of the 10 sites (electronic supplementary material, figure S7). This allows us to account for some sites being edited faster or slower than others and hence refine the estimate of the trees’ branch lengths. On average, the median tree height under setting c is 1.25 time units larger (i.e. further in the past) compared to setting a and b (see electronic supplementary material, figure S8). Intuitively, a larger tree height allows more time for the same number of cell divisions, which explains the reduced cell division rate.

In our final analysis, we assess how much information the sequences provide about the cell division and death rate. To this end, we perform a Bayesian analysis without the sequence data, effectively leaving the number of cells present at the time of sequencing as input data to estimate the cell division and death rate (electronic supplementary material, figure S10). Compared to the analyses including the sequencing data (but otherwise using the same priors), the posterior of the cell division rate (95% HPD [0.02, 0.3] compared to [0.07, 0.09] with sequence data) and death rate (95% HPD [0.02, 0.3] compared to [0.03, 0.05] with sequence data) are 10 times broader, making their estimates 10 times less precise. This shows that the sequencing data provides valuable information about the population dynamic parameters.

## 4. Discussion

In this work, we introduced a new framework to estimate single-cell trees and, for the first time, population dynamic parameters from genetic lineage tracing data. We first developed a time-dependent editing model and then derived its likelihood calculation. Finally, we implemented the likelihood within a Bayesian MCMC framework, enabling co-estimation of time-scaled single-cell trees, parameters of the editing model, and population dynamic parameters from genetic lineage tracing data. After validating our implementation via simulations, we additionally demonstrated that incorporating prior information and experimental replicates can further improve TiDeTree’s power. In all, our TiDeTree framework will enable new insights on cell population dynamics during the development of organisms and tissues. TiDeTree is a timely contribution to the field of developmental biology, as evidenced by the fact that TiDeTree’s release is concurrent with another maximum-likelihood approach [21]. In comparison, TiDeTree is available within the widely used BEAST 2 platform for Bayesian phylodynamic inference [14]. This allows access to an ever-growing array of clock models, tree priors and other models. This provides users of TiDeTree immense flexibility to set up analyses that best fit their model systems. Frameworks for model selection by estimating the marginal likelihood [22,23] and evaluating absolute model fit by posterior predictive simulation [24,25] are also available and can be easily used in combination with our model. For instance, we assumed a strict clock model in our analyses, and thus assume that the editing rate does not vary over the short experimental time span. However, for longer time spans (e.g. during ontogenesis), the editing rate may vary over time. To account for this, relaxed clock models are readily available in BEAST 2.

Given that TiDeTree is the first Bayesian phylodynamic framework for inferring cell population dynamics from genetic lineage tracing data, we additionally explored the information content in these datasets for such inference. Namely, we investigated how many population dynamic parameters we can concurrently estimate. [26] showed that under birth-death sampling models as used here, at most two out of the three parameters (cell division rate, apoptosis rate and sampling proportion) can be obtained from a reconstructed tree. We first evaluated the information content of the data based on our simulations where editing occurred for half of the experimental time span. In that setting, only one parameter of a birth–death sampling model could be estimated from a single alignment. However, when we added additional information, e.g. by assuming scarring rates are known or by additionally adding experimental replicates, we could improve the accuracy, coverage and precision of the parameter estimates. Our analysis of an experimental lineage tracing dataset [15] supported this finding. Here, we could infer two parameters, the cell division and death rate, by pooling them across 106 experimental replicates. We showed that the lineage tracing data, albeit noisy, provided the necessary signal to estimate the cell population dynamics.

These results underscore two possible routes to further improving the signal for estimation of cell population dynamics from genetic lineage tracing data. First, we can increase the signal contained within individual trees, e.g. by increasing the number of targets (which correspond to the sites in the phylogenetic likelihood) or increasing the editing duration, e.g. by using repeatedly editing homing CRISPR barcode systems [27] or insertion based recorders [9]. Second, we can include more experimental replicates and develop an approach through which they can inform complementary time spans (for instance, replicates 1–10 inform the first time span and replicates 10–20 inform second time span, etc.). These suggestions for experimental design could further improve TiDeTree’s power.

As a current benchmark, we compared TiDeTree to several recently published alternative methods using benchmarks introduced by [10,11]. We show that TiDeTree performs among the top three methods for tree topology inference (figure 5). In addition, TiDeTree has several unique features not captured by the benchmark. First, since TiDeTree is implemented in a Bayesian framework, it generates posterior distributions of plausible trees and model parameters. In comparison, current alternative methods generate an estimate for the single best tree. Thus, TiDeTree enables direct assessment of uncertainty. Next, TiDeTree estimates time-scaled trees. As the method in [12], TiDeTree assumes by default a constant editing rate (a so-called molecular clock assumption) in order to timescale the tree. However, while their method can only estimate a relative ordering along parallel lineages, TiDeTree estimates time-scaled trees, i.e. trees with branch lengths corresponding to the absolute time interval between cell divisions. Such time-scaled cell phylogenies will allow developmental biologists to address questions on the rates and timing of developmental events using lineage tracing data. Finally, compared to other methods, TiDeTree can estimate additional parameters of interest (e.g. the scarring rates, clock rate, and scarring onset). This could lead to model overparameterization; however, the increased complexity of our model is directly tied to the experimental reality and will hence better capture the specific features of this substitution process. In fact, there is evidence that underparameterized substitution models are more likely to lead to biased inference than overparameterized ones in Bayesian phylogenetics [28,29]. In summary, TiDeTree performs comparably in terms of accuracy to state-of-the-art methods for cell lineage tree topology estimation while additionally enabling direct assessment of uncertainty, estimation of a time-scaled tree, and co-estimation of additional parameters.

Despite these advantages, TiDeTree has several statistical and computational limitations. A limitation of our framework is the comparatively long run time, which results primarily from estimating the posterior distribution of all parameters and trees. In particular, evaluating the posterior probability of subtrees with identical sequences is time-intensive. Compared to the popular method Cassiopeia [10], which computes a single best lineage tree of 500 cells in approximately 3 h, the current TiDeTree framework requires an average of 400 h (electronic supplementary material, figure S3) to compute the posterior distribution over all parameters. We expect that the greatest speedup can be achieved by developing a more efficient method for sampling subtrees with identical sequences in TiDeTree’s implementation, because the MCMC sampling algorithm faces difficulties navigating the low-likelihood valleys. Employing sampling schemes that do not estimate the posterior distribution of all parameters will also result in a significant reduction in run time. However, given the limited signal of most lineage recording systems and the possibility of recurrent scarring events, we argue that taking into account the probabilistic uncertainty as done in TiDeTree is crucial to avoid overly confident conclusions. Thus, computational speed-ups to TiDeTree will be a key area of future development.

Finally, the TiDeTree framework opens the door to new applications and new research questions in developmental biology. In particular, a promising future direction for TiDeTree will be multi-type analyses. Given annotated cell types for sampled cells (possibly based on single-cell RNA-seq data), TiDeTree in conjunction with the multi-type model in BEAST 2 [30] can in principle estimate cell differentiation rates, as well as cell-type-specific division and apoptosis rates [1]. Such parameters quantify core processes of developmental biology. We view our methodology as a basis for Bayesian inference of single-cell time trees in conjunction with developmental parameters. Thus, we see great promise that the advances in single-cell lineage tracing technology combined with advances in single-cell phylodynamic methodology will greatly enhance our understanding of developmental processes.

## Data Availability

The source code of the BEAST 2 package TiDeTree is available at https://github.com/seidels/tidetree. The code to perform the analyses and generate the figures can be found at https://github.com/seidels/tidetree-material. All data underlying the analyses are available from the Dryad Digital Repository: https://doi.org/10.5061/dryad.qz612jmk7 [31]. Supplementary material is available online [32].
