# Correction to: ‘TiDeTree: a Bayesian phylogenetic framework to estimate single-cell trees and population dynamic parameters from genetic lineage tracing data’ (2022) by Seidel & Stadler

**DOI:** 10.1098/rspb.2022.2312

**Published:** 2023-01-11

**Authors:** Sophie Seidel, Tanja Stadler

*Proc. R. Soc. B* **289**, 20221844. (Published online 9 November 2022). (https://doi.org/10.1098/rspb.2022.1844)

We would like to submit the following correction.

In figure 5*a*, the axis labels have to be exchanged, meaning that ‘mean triplet distance’ should show on the *y*-axis and ‘mean RF distance’ should show on the *x*-axis.

This has now been corrected on the publisher's website.

